# Giant Primary Hepatic Endodermal Sinus Tumor: Multidisciplinary Management and Long‐Term Survival

**DOI:** 10.1155/crom/8838504

**Published:** 2026-01-10

**Authors:** Katia Roque, Rossana Ruiz, Renier Cruz, Andrea Ramirez-Aramburú, Eloy Ruiz, Carlos Castaneda, Marco Galvez-Nino, Ofelia Coanqui, Natalia Valdiviezo, Mivael Olivera Hurtado de Mendoza, Ramon Andrade de Mello, Ilaria Colombo, Luis Mas

**Affiliations:** ^1^ Department of Medical Oncology, National Institute of Neoplastic Diseases (INEN), Lima, Peru; ^2^ Nove de Julho University (UNINOVE), São Paulo, Brazil, uninove.br; ^3^ Faculty of Human Medicine, National University of San Marcos, Lima, Peru, unmsm.edu.pe; ^4^ Department of Pathology, National Institute of Neoplastic Diseases (INEN), Lima, Peru; ^5^ Faculty of Human Medicine, Cayetano Heredia Peruvian University, Lima, Peru, cayetano.edu.pe; ^6^ Department of Oncologic Abdominal Surgery, National Institute of Neoplastic Diseases (INEN), Lima, Peru; ^7^ Faculty of Human Medicine, Universidad Científica del Sur, Lima, Peru, cientifica.edu.pe; ^8^ Department of Medical Oncology, Albert Einstein Israeli Hospital, São Paulo, Brazil; ^9^ Master’s Programme in Medical Oncology, University of Buckingham, Buckingham, UK, buckingham.ac.uk; ^10^ Department of Medical Oncology, Oncology Institute of Southern Switzerland (IOSI), Bellinzona, Switzerland, ior.iosi.ch

**Keywords:** alpha-fetoprotein, case report, extragonadal germ cell, primary hepatic endodermal sinus tumor

## Abstract

The endodermal sinus tumor (EST), also known as yolk sac tumor, accounts for 20% of germ cell tumor cases, typically occurring in gonadal locations. However, 1%–5% can present with an extragonadal localization. Primary hepatic EST is an extremely rare entity and poses a diagnostic challenge for the appropriate management of this pathology. We present the case of a 34‐year‐old woman who presented with a single hepatic mass associated with elevated alpha‐fetoprotein (AFP) levels. Initially, hepatocellular carcinoma (HCC) was suspected, leading to a right hepatectomy, which resulted in pathology findings consistent with an EST. Following surgery, the patient underwent four courses of BEP chemotherapy, showing a partial response with residual lesions. The patient received two more courses of EP chemotherapy, with a PET CT showing a complete response. At over 5 years of follow‐up, the patient remains clinically stable, with negative tumor markers, no evidence of disease, and leading a normal life. Primary hepatic EST is an infrequent but important differential diagnosis of HCC, particularly in young women without cirrhosis who present with markedly elevated AFP levels. Early biopsy confirmation and multidisciplinary management are essential, as this chemosensitive tumor may achieve long‐term survival with timely systemic treatment.

## 1. Introduction

Endodermal sinus tumors (ESTs), also known as yolk sac tumors, represent 20% of germ cell tumor cases, typically arise in the ovaries, and present with a pelvic mass associated with elevated alpha‐fetoprotein (AFP) levels. They usually occur in children and young adults, with an average age of 16–20 years [1]. However, 1%–5% of germ cell tumors develop at extragonadal sites; in the case of EST, this occurs in up to 10% of cases. The most common extragonadal locations are the anterior mediastinum, retroperitoneum, sacrococcygeal region, and central nervous system [2–6]. Primary hepatic EST is an extremely rare entity, posing both diagnostic and therapeutic challenges in the management of this pathology.

## 2. Case Presentation

In May 2019, a 34‐year‐old female patient, without significant personal or family medical history, presented with a 6‐month history of abdominal distension and persistent pain in the right upper quadrant. Physical examination revealed marked hepatomegaly, with a palpable mass approximately 8 cm below the costal margin extending into the right flank. There was no evidence of jaundice or stigmata of chronic liver disease. Initial laboratory studies showed a normal blood count and slight elevation of aspartate aminotransferase (AST) (92 U/L), alkaline phosphatase (ALP) (142 U/L), and gamma‐glutamyl transferase (GGT) (93 U/L). Alanine aminotransferase (ALT), prothrombin time (PT), and serum bilirubin (BT) levels were within normal limits. AFP (352,030 ng/mL) and lactate dehydrogenase (LDH) (2232) levels were significantly elevated, while other tumor markers, including CA 19‐9 and CEA, showed no abnormalities. Hepatitis B and C serologies were negative. An abdominal contrast‐enhanced CT scan showed an extensive neoplastic lesion with areas of hypervascular enhancement and “washout,” involving Segments VIII, VII, and VI and part of Segments I, V, and IV, causing hepatomegaly and deforming the right hemidiaphragm with suspected diaphragmatic and right pleural infiltration. Additionally, a thoracic CT showed nodular lesions in the right lower lung lobe (Figure 1).

Figure 1Chest and abdominal CT scans at diagnosis. (a) Contrast‐enhanced CT of the chest. (b) Axial CT image of the abdomen showing a lobulated tumor measuring 50 × 30 cm involving Segments I, IV, V, VI, VII, and VIII. (c) Coronal CT image of the abdomen demonstrating capsular rupture and diaphragmatic infiltration in Segments IVA and VIII. CT: computed tomography.

**Figure:**
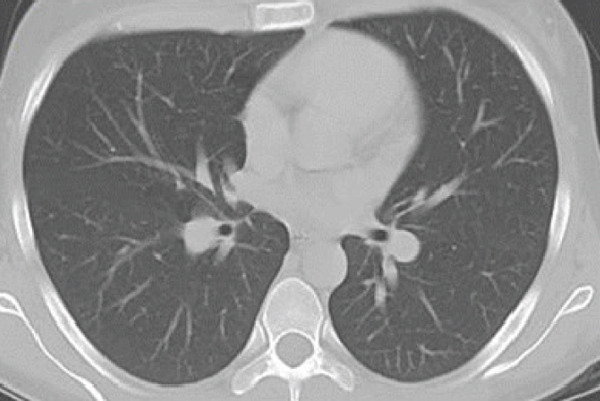
(a)

**Figure:**
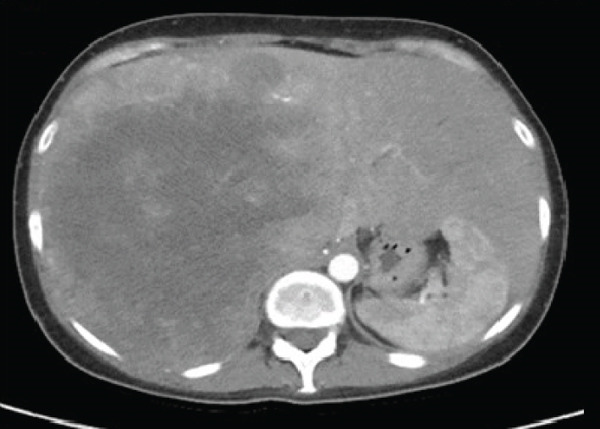
(b)

**Figure:**
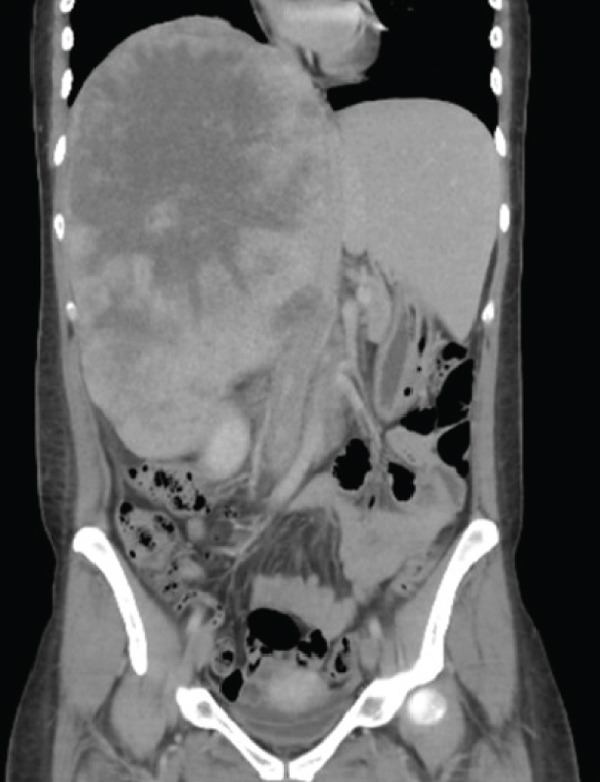
(c)

Based on the clinical, laboratory, and imaging findings, the suspected diagnosis was metastatic hepatocellular carcinoma (HCC), and palliative surgery was planned. No biopsy was performed, as in this clinical context, a definitive diagnosis of HCC can be established solely on the basis of characteristic imaging findings.

The patient underwent an exploratory laparotomy with right hemi hepatectomy, with intraoperative findings describing a 50 × 30‐cm lobulated tumor involving the hepatic surface and parenchyma in Segments I, IV, V, VI, VII, and VIII, with capsular rupture and diaphragmatic infiltration at Segments IVA and VIII. Additionally, nodules were identified in the omentum, all of which were resected, leaving only macroscopic residual disease in the diaphragm. In the immediate postoperative period, the patient developed hypovolemic shock secondary to intraoperative bleeding, requiring vasopressor support in the intermediate care unit with favorable evolution. The pathology report was compatible with primary hepatic EST, with positive margins, lymphovascular invasion (+), and immunohistochemistry showing glypican‐3 (+), AFP (+), and Hep‐Par‐1 (−) (Figure 2). Chorionic gonadotropin hormone was within normal limits (hCG 0.48), and transvaginal ultrasound ruled out a probable adnexal primary lesion. Postoperatively, the laboratory showed a normal liver profile and a marked decrease in AFP (43,388 ng/mL) and LDH (663 U/L).

Figure 2Macroscopic and microscopic pathological findings. (a) Gross appearance of the resected liver tumor specimen. (b) Histopathological features of the liver tumor on hematoxylin–eosin staining (original magnification ×100). (c) Schiller–Duval bodies (original magnification ×100). Immunohistochemical staining showing (d) AFP‐positive, (e) Glypican‐3‐positive, and (f) HepPar‐1‐negative expression.

**Figure:**
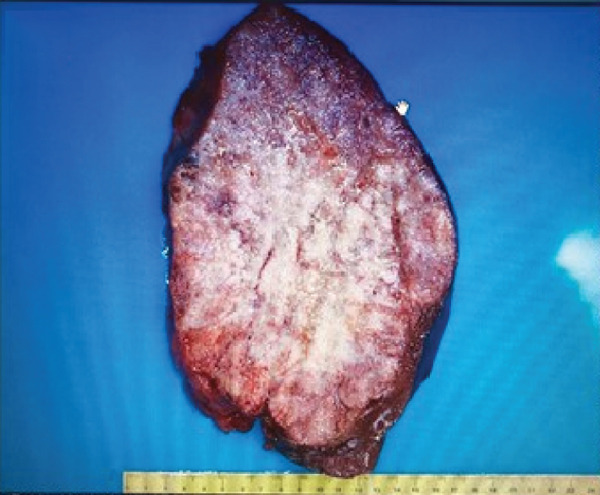
(a)

**Figure:**
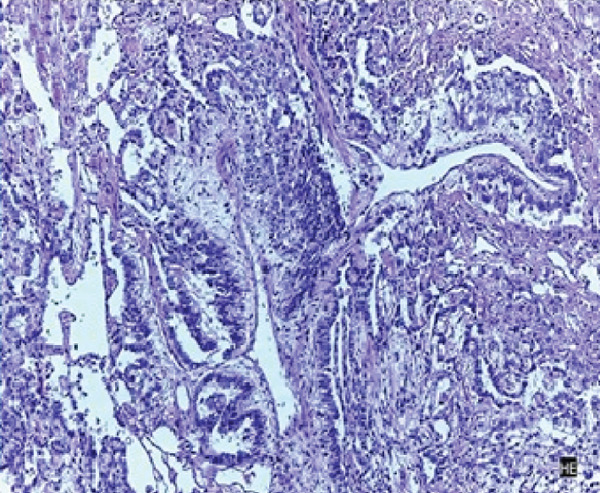
(b)

**Figure:**
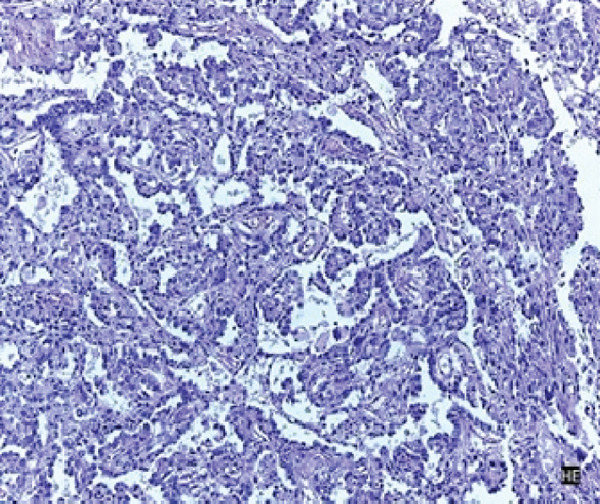
(c)

**Figure:**
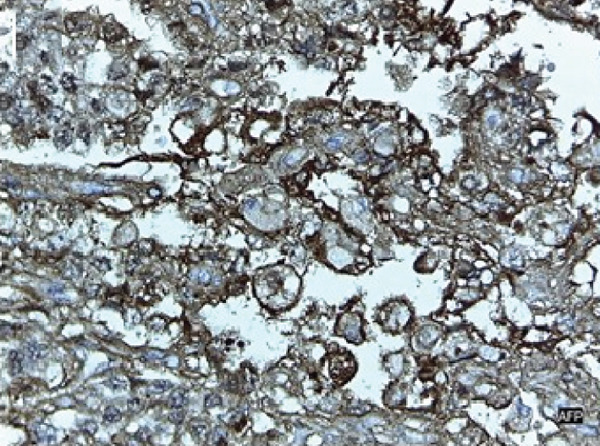
(d)

**Figure:**
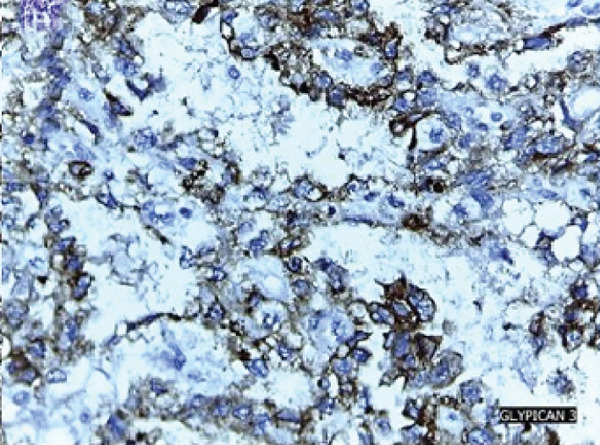
(e)

**Figure:**
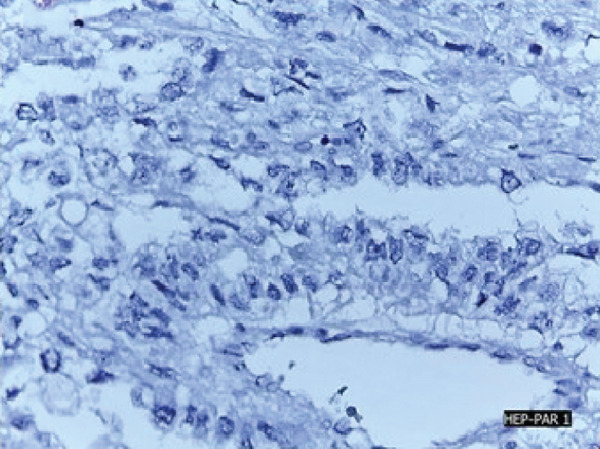
(f)

One‐month postoperative CT scans showed new hepatic lesions involving Segments II and III, with the largest measuring 36 mm, as well as superior mesenteric lymphadenopathy, multiple right subphrenic infiltrative lesions associated with ascites, and alteration of fatty planes. Thoracic CT confirmed right adjacent diaphragmatic involvement and persistent lung nodules (Figure 3).

Figure 3One‐month postoperative chest and abdominal computed tomography (CT) scans. (a) Contrast‐enhanced CT of the chest demonstrating right pleural effusion. (b) Axial CT image of the abdomen showing new hepatic lesions involving Segments II and III, with the largest measuring 36 mm. (c) Coronal CT image of the abdomen demonstrating superior mesenteric lymphadenopathy, multiple right subphrenic infiltrative lesions, ascites, and alteration of fat planes.

**Figure:**
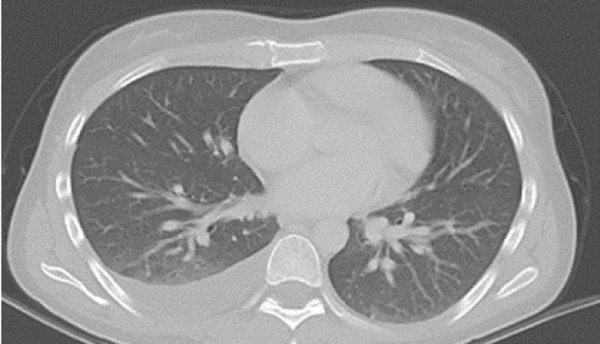
(a)

**Figure:**
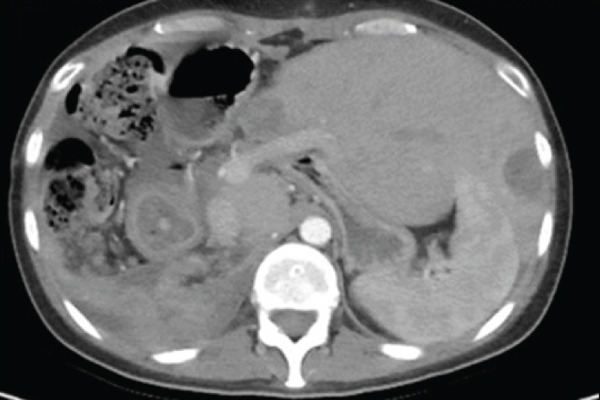
(b)

**Figure:**
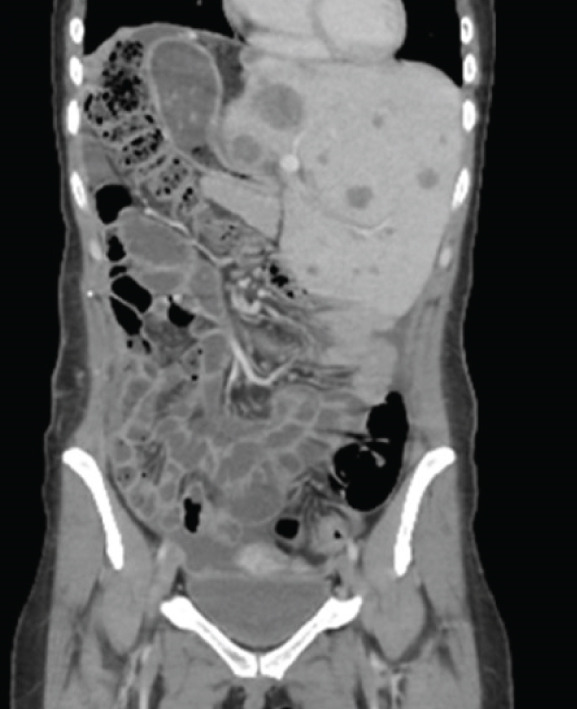
(c)

Given the tumor size, the surgical findings, residual disease, and rapid progression, this case corresponds to an advanced‐stage primary hepatic EST. According to the International Germ Cell Cancer Collaborative Group (IGCCCG) classification, the markedly elevated AFP (> 1000 ng/mL) and extragonadal location place the patient in the poor prognosis group. The patient initially received four cycles of chemotherapy with the BEP regimen (bleomycin/etoposide/cisplatin) from July to October 2019, with adequate adherence and good tolerance. Posttreatment imaging demonstrated a partial response (thoracic CT showed a single subpleural nodule, while abdominal CT demonstrated a reduction in the number and size of hepatic lesions compared to the previous study, with the largest lesion reported in Segment II, measuring 14 × 23 mm) and negative tumor markers (AFP, 7.12; hCG, 0.12; and LDH, 211).

The patient was evaluated for both abdominal and thoracic surgery. Although the subpleural lesion was resectable, multiple hepatic lesions precluded additional surgical intervention. She therefore received two additional cycles of EP (etoposide/cisplatin), administered due to concerns regarding cumulative bleomycin toxicity (total cumulative bleomycin dose = 360 U). Follow‐up imaging showed residual liver lesions (largest: 9 mm, no diffusion restriction) and no pulmonary lesions, with negative tumor markers (Figure 4). A multidisciplinary team recommended a PET‐CT, which revealed a subpleural nodule and small hepatic lesions without hypermetabolism, suggesting sequelae rather than active disease. At over 5 years of follow‐up, the patient remains free of recurrence and has been leading a normal life.

Figure 4Postchemotherapy chest and abdominal computed tomography (CT) scans. (a) Contrast‐enhanced CT of the chest demonstrating no detectable lesions. (b) Axial CT image of the abdomen showing increased volume of the left hepatic lobe and caudate lobe, with hypodense residual nodules measuring < 10 mm. (c) Coronal CT image of the abdomen demonstrating post–right hepatectomy status and absence of lymphadenopathy.

**Figure:**
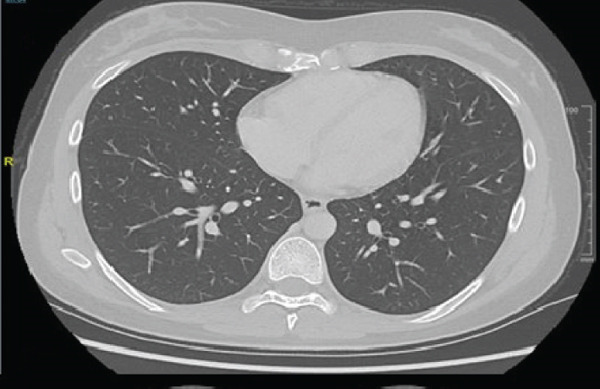
(a)

**Figure:**
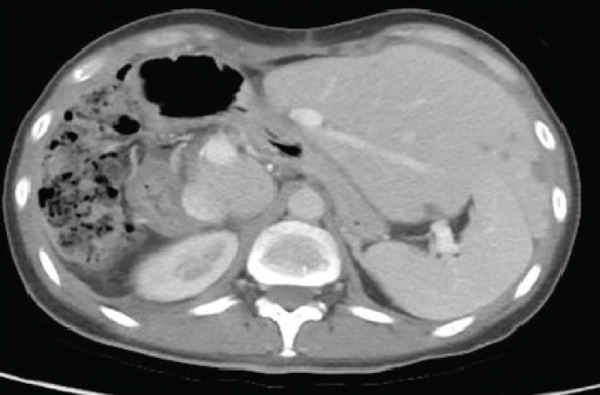
(b)

**Figure:**
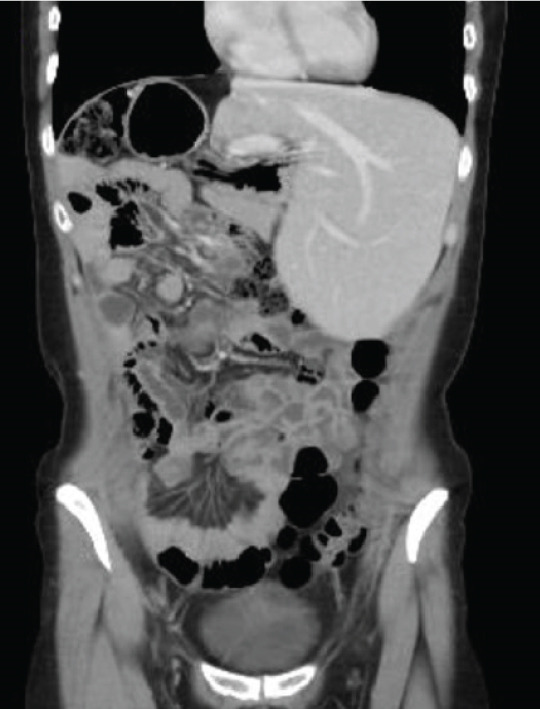
(c)

## 3. Discussion

The extragonadal presentation of EST is very infrequent, though about 10%–15% develop in extragonadal regions such as the mediastinum, retroperitoneum, sacrococcygeal area, and bladder, among others [7, 8]. They have been predominantly reported in children and only a few cases in adulthood [9]. A prominent hypothesis for their extragonadal presence is that these tumors originate from germ cells that deviated from their typical migration path from the yolk sac to the genital ridge during embryogenesis. Another theory suggests that these tumors arise from pluripotent embryonic cells that did not undergo differentiation during liver embryogenesis [2, 10].

Fewer than 20 cases of primary hepatic EST in adults have been reported in the literature (Table 1), most of them with long‐term poor prognosis [2, 11–13, 17]. These tumors may be solitary or multiple, usually located on the right side of the liver, and can grow significantly large (up to 20 cm). Macroscopically, they appear fleshy and gray or brownish in color and are solid or cystic when sectioned, sometimes displaying a honeycomb pattern [2, 10, 26, 28].

**Table 1:** Reviewed of reported cases of primary endodermal sinus tumor of the liver in adults.

**Author**	**Year of publication**	**Age at diagnosis (years old)**	**Sex**	**AFP (ng/mL)**	**Size of tumor (cm)**	**Number of lesions**	**Localization**	**Type of surgery**	**Systemic treatment or other**	**Overall survival**	**Status**
Yan [11]	1982	58	M	330,000	Extensive tumor	Multiple	Both lobes	NA	NA	NA	NA
Natori et al. [12]	1983	29	F	4270	17 × 15 × 10	Single	Right lobe	Repeated hepatectomy	Postoperative chemotherapy: Actinomycin D, vincristine, and cyclophosphamide	6.6 months	Dead
Narita et al. [13]	1988	27	F	28,500	11 × 11 × 9.5	Single	Right lobe	Partial hepatectomy	Postoperative chemotherapy: Cisplatin, adriamycin, and pepleomycin followed by maintenance therapy	1 year	Dead
Villaschi and Balistreri [14]	1991	28	F	500	15 × 15 × 15	Single	Left lobe	Partial hepatectomy	NA	NA	NA
Whelan et al. [15]	1992	27	F	89,000	15 × 15	Single	Right lobe	Right hemihepatectomy	Preoperative chemotherapy BEP	5+ years	Alive
Higuchi and Kikuchi [16]	1993	24	F	115,500	Large tumor	Multiple	Right lobe	None	TACE	NA	NA
Wong et al. [17]	1998	28	F	14,614	15 × 15 × 6	Single	Right lobe	Partial hepatectomy	None	1 year	Alive
Gunawardena et al. [18]	2002	37	M	326	10 D	Single	Left lobe	Partial hepatectomy	NA	NA	NA
Toumi et al. [19]	2004	28	F	54,000	13.7 D	Single	Right lobe	Right hepatectomy	Postoperative chemotherapy BEP, followed by second‐line salvage chemotherapy: Vinblastine, ifosfamide, and cisplatin+high‐dose chemotherapy	NA	NA
Lenci et al. [2]	2008	64	M	400	2.8 D	Single	Right lobe	None	Laser ablation+TACE+liver transplantation	12+ months	Alive
Hosala et al. [20]	2014	20	F	> 71,753	15 × 15 × 12	Single	Right lobe	Right hemicolectomy	Postoperative chemotherapy BEP	4+ years	Alive
Reznichenko et al. [21]	2016	39	F	34.32	25 D	Multiple	Both lobes	Right extended hepatectomy	NA	NA	NA
Evers et al. [22]	2018	45	F	> 10,000	11.3 × 9.5 × 7.4	Multiple	Right lobe	Partial hepatectomy	Preoperative chemotherapy BEP	NA	NA
Prilutskiy et al. [23]	2018	56	F	505.6	13 D	Single	Right lobe	Partial hepatectomy	Preoperative chemotherapy: EP	NA	NA
Vanidassane et al. [24]	2019	27	M	120,000	13.6 × 10.6 × 10.6	Single	Both lobes	None	BEP+TIP+gemcitabine and oxaliplatin	NA	NA
Jindal et al. [25]	2021	28	F	55,897	NA	Multiple	Right lobe	None	BEP	NA	NA
Liu et al. [26]	2023	31	F	31,132	14.4 × 11.8 × 10.2	Multiple	Both lobes+metastasis of the greater omentum	Preoperative TACE+liver transplantation and omentectomy	Postoperative chemotherapy BEP+larotrectinib	34+ months	Alive
Patel et al. [27]	2024	32	M	> 1000	NA	Single	Enlarged periportal and periductal lymph nodes	None	BEP+bone marrow transplantation	12+ months	Alive
Roque^a^	2025	34	F	352,030	50 × 30	Single	Right lobe+diaphragmatic infiltration	Right extended hepatectomy+omentectomy	Postoperative chemotherapy BEP+2 additional cycles of chemotherapy EP	5+ years	Alive

Abbreviations: BEP, bleomycin, etoposide, and cisplatin; EP, etoposide and cisplatin; NA, not available; TACE, transcatheter arterial embolization; TIP, paclitaxel, ifosfamide, and cisplatin.

^a^This study.

Microscopically, ESTs exhibit several characteristic patterns, including Schiller–Duval bodies, which are papillae containing a central blood vessel, as well as elongated or festooned cord patterns. Cytologically, these tumors show eosinophilic hyaline globules. Immunohistochemically, they test positive for AFP in patches, PLAP, SALL‐4, and Glypican‐3, while Hep‐Par 1 is typically negative except in the hepatoid variant. Histologically, although the embryonal type of hepatoblastoma frequently presents primitive cells that may resemble those in EST, they lack Schiller–Duval bodies and cytoplasmic eosinophilic hyaline bodies [2, 10, 14, 26].

This case involves a young woman with no identifiable risk factors who presented with a solitary hepatic tumor and elevated AFP levels. Imaging studies revealed a vascularized mass with “washout” characteristics, leading to the decision to forgo a preoperative needle biopsy. Given the clinical presentation, tumor marker elevation, and imaging findings, the primary differential diagnosis at the time of surgery was HCC. This consideration was further supported by local epidemiological data, which indicate that most patients with HCC in our setting are young and noncirrhotic and present with large hepatic masses [29].

Although HCC and EST share overlapping features, they differ in several clinically meaningful ways that are essential for accurate diagnosis. Both tumors may present as large hepatic masses with markedly elevated AFP levels. However, HCC typically occurs in older individuals with underlying cirrhosis or chronic hepatitis, whereas EST predominantly affects younger patients without preexisting liver disease. Imaging findings may be misleading, as both tumors can demonstrate hypervascularity with washout on contrast‐enhanced studies, underscoring the limited specificity of radiology in differentiating between them. Histologically, however, the distinction becomes clear. EST is characterized by hallmark features such as Schiller–Duval bodies, eosinophilic hyaline globules, and strong immunopositivity for AFP, SALL‐4, and Glypican‐3, along with the absence of Hep‐Par 1 expression. In contrast, HCC demonstrates hepatocellular differentiation and typically retains Hep‐Par 1 positivity [10, 28, 30].

Recognizing these diagnostic differences is essential to avoid misclassification and ensure appropriate therapeutic decision‐making. While surgery is the primary treatment for resectable HCC, EST is a highly chemosensitive germ cell tumor in which platinum‐based chemotherapy is the cornerstone of management. Misdiagnosis of EST as HCC may therefore lead to unnecessary major hepatic resections, delayed initiation of effective systemic therapy, and potentially poorer outcomes. Conversely, timely recognition of EST allows prompt administration of germ cell–directed chemotherapy, which is associated with high response rates and the potential for long‐term survival, even in cases with significant tumor burden.

Therefore, EST should be included in the differential diagnosis of hepatic masses in young patients without risk factors for HCC, particularly when AFP levels are disproportionately elevated [21]. According to a report by Wong et al., which describes seven cases of primary hepatic EST in adults, it is recommended that in female patients aged 25–30 years with a cystic hepatic tumor, without signs of liver cirrhosis, and AFP levels exceeding 3000, an EST should be suspected, warranting a confirmatory biopsy [10, 17, 31].

The current treatment for EST is chemotherapy based on platinum salts, with the BEP regimen being administered over four cycles every 3 weeks. This regimen has a high response rate and survival outcomes (85% 5‐year survival rate). Surgery is also an option in some cases [20]. Follow‐up should involve periodic monitoring of AFP and hCG levels every 2–4 months if they were elevated at diagnosis. For patients with residual disease indicated by imaging but normal AFP and hCG levels, surgery or observation is a feasible option. However, if residual disease is accompanied by elevated tumor markers, a second‐line chemotherapy regimen, such as TIP (paclitaxel/ifosfamide/cisplatin) for two courses, or high‐dose chemotherapy with the possibility of autologous stem cell transplantation should be considered [1, 32–34].

Given the initial diagnosis of HCC, the initial management included a right hepatectomy, considering the tumor size and previously reported survival outcomes in our population [15]. However, following the pathological findings, systemic treatment was proposed. Imaging before treatment revealed new hepatic lesions and ascites, which required peritoneal drainage on two occasions, highlighting the aggressive nature of the disease. It is important to note that in the management of primary hepatic EST, surgery does not play a primary role, even when complete resection with free margins is achieved. Nevertheless, surgical resection remains a key component of treatment in selected cases of giant primary hepatic EST, ideally following tumor downstaging with chemotherapy. Multidisciplinary coordination is essential for optimizing outcomes.

The favorable response to chemotherapy further supported the diagnosis of a germ cell tumor, with a complete response achieved postchemotherapy, as confirmed by PET‐CT imaging. Follow‐up was conducted with AFP monitoring every 3 months. However, during the COVID‐19 pandemic, the patient was lost to follow‐up for 6 months. Upon returning, control imaging and tumor marker assessments were performed, all of which were negative. This is the first report in the country of a very rare, high‐volume disease where multidisciplinary management led to long‐term survival.

In summary, primary hepatic EST is an extremely rare condition but should be considered as a differential diagnosis to HCC, particularly in young female patients without hepatitis infection or signs of liver cirrhosis and with elevated AFP levels [18]. In such cases, a liver biopsy should be considered, as this chemosensitive tumor can achieve favorable outcomes. Accurate diagnosis and multidisciplinary management are crucial for guiding therapeutic decisions and ensuring appropriate treatment.

## Ethics Statement

This case report does not contain any personal identifiers or images that could compromise the anonymity of the patient. Nevertheless, written informed consent was obtained from the patient for publication of this case report and any accompanying images.

## Disclosure

All authors have read and approved the final version of the manuscript.

## Conflicts of Interest

The authors declare no conflicts of interest.

## Author Contributions

Conceptualization: Katia Roque. Investigation (clinical management and data collection): Katia Roque, Rossana Ruiz, Renier Cruz, Eloy Ruiz, Carlos Castaneda, Marco Galvez‐Nino, Ofelia Coanqui, Natalia Valdiviezo, Mivael Olivera Hurtado de Mendoza, and Luis Mas. Methodology: Katia Roque, Andrea Ramirez‐Aramburú, Eloy Ruiz, Carlos Castaneda, and Marco Galvez‐Nino. Project administration: Ofelia Coanqui, Natalia Valdiviezo, and Mivael Olivera Hurtado de Mendoza. Resources: Renier Cruz, Eloy Ruiz, and Mivael Olivera Hurtado de Mendoza. Supervision: Carlos Castaneda, Ramon Andrade de Mello, Ilaria Colombo, and Luis Mas. Visualization: Andrea Ramirez‐Aramburú. Writing—original draft: Katia Roque, Rossana Ruiz, Renier Cruz, and Andrea Ramirez‐Aramburú. Writing—review and editing: Carlos Castaneda, Ramon Andrade de Mello, Ilaria Colombo, and Luis Mas.

## Funding

No funding was received for this manuscript.

## Data Availability

The data that support the findings of this study are available from the corresponding author upon reasonable request.
